# *catena*-Poly[silver(I)-bis­[μ-4-methyl-1*H*-1,2,4-triazole-3(4*H*)-thione-κ^2^ *S*:*S*]-silver(I)-di-μ-thio­cyanato-κ^2^ *S*:*N*;κ^2^ *N*:*S*]

**DOI:** 10.1107/S160053681300946X

**Published:** 2013-04-13

**Authors:** Kultida Kodcharat, Chaveng Pakawatchai, Saowanit Saithong

**Affiliations:** a Department of Chemistry and Center of Excellence for Innovation in Chemistry, Faculty of Science, Prince of Songkla University, Hat Yai, Songkhla 90112, Thailand

## Abstract

In the title one-dimensional coordination polymer, [Ag_2_(NCS)_2_(C_3_H_5_N_3_S)_2_]_*n*_, the Ag^I^ atom adopts a distorted tetra­hedral AgNS_3_ geometry. Adjacent Ag^I^ atoms in the [001] chain are alternately linked by pairs of bridging 4-methyl-1*H*-1,2,4-triazole-3(4*H*)-thione (Hmptrz) ligands (*via* their S atoms) and double thio­cyanate bridges linking through both S and N atoms (μ-1,3-SCN). An intra­chain N—H⋯N hydrogen bond occurs between the NH group of the triazole ring and the N atom of the thio­cyanate bridging ligand. A (101) sheet structure arises from inter­chain S⋯N short contacts [3.239 (3) Å] involving the thio­cyanate S atom and the triazole-ring N atom and possible very weak π–π stacking [centroid–centroid separation = 4.0762 (18) Å] between the triazole rings.

## Related literature

For examples of complexes with multifunctional ligand donors, see: Zhang *et al.*(2009 ▶); Wang *et al.* (2011 ▶). For background to complexes containing derivatives of the 1,2,4-triazole ligand, see: Zhang *et al.* (1999 ▶); Jiang *et al.* (2011 ▶). For the thio­cyanate bridging ligand, end-to-end mode, see: Vicente *et al.* (1997 ▶); Chen *et al.* (1999 ▶); Diaz *et al.* (1999 ▶); Goher *et al.* (2000 ▶); Song *et al.* (2000 ▶); Cai *et al.* (2007 ▶); Saithong *et al.* (2007 ▶).
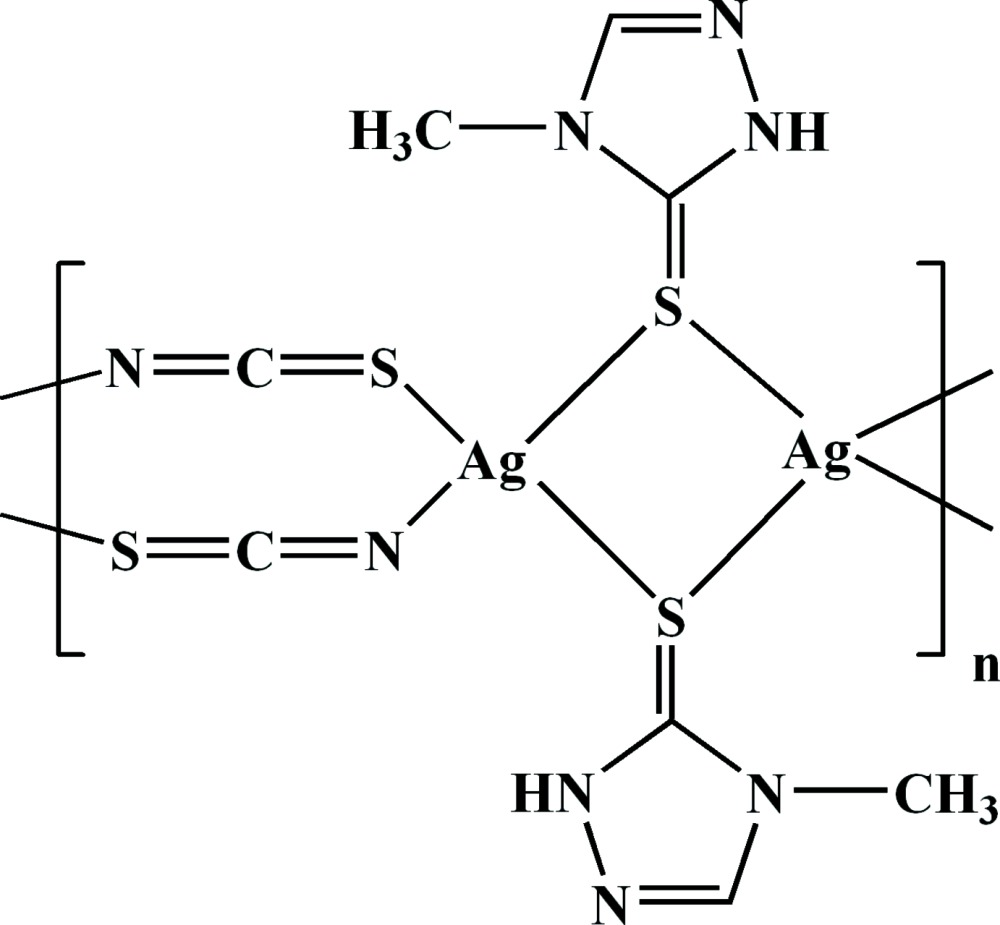


## Experimental

### 

#### Crystal data

[Ag_2_(NCS)_2_(C_3_H_5_N_3_S)_2_]
*M* *_r_* = 562.22
Triclinic, 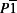

*a* = 7.4842 (6) Å
*b* = 7.5420 (6) Å
*c* = 8.4262 (7) Å
α = 79.985 (2)°
β = 84.329 (2)°
γ = 64.508 (1)°
*V* = 422.62 (6) Å^3^
*Z* = 1
Mo *K*α radiation
μ = 2.82 mm^−1^
*T* = 293 K
0.31 × 0.12 × 0.05 mm

#### Data collection

Bruker APEX CCD diffractometer
Absorption correction: multi-scan (*SADABS*; Bruker, 2003 ▶) *T*_min_ = 0.682, *T*_max_ = 0.879
5887 measured reflections
2083 independent reflections
1904 reflections with *I* > 2σ(*I*)
*R*_int_ = 0.026

#### Refinement

*R*[*F* ^2^ > 2σ(*F* ^2^)] = 0.029
*wR*(*F* ^2^) = 0.070
*S* = 1.05
2083 reflections
104 parameters
1 restraint
H atoms treated by a mixture of independent and constrained refinement
Δρ_max_ = 0.79 e Å^−3^
Δρ_min_ = −0.63 e Å^−3^

### 

Data collection: *SMART* (Bruker, 2003 ▶); cell refinement: *SAINT* (Bruker, 2003 ▶); data reduction: *SAINT*; program(s) used to solve structure: *SHELXS97* (Sheldrick, 2008 ▶); program(s) used to refine structure: *SHELXL97* (Sheldrick, 2008 ▶); molecular graphics: *Mercury* (Macrae *et al.*, 2008 ▶); software used to prepare material for publication: *SHELXTL* (Sheldrick, 2008 ▶), *PLATON* (Spek, 2009 ▶) and *publCIF* (Westrip, 2010 ▶).

## Supplementary Material

Crystal structure: contains datablock(s) I, global. DOI: 10.1107/S160053681300946X/hb7066sup1.cif

Structure factors: contains datablock(s) I. DOI: 10.1107/S160053681300946X/hb7066Isup2.hkl

Supplementary material file. DOI: 10.1107/S160053681300946X/hb7066Isup3.mol

Supplementary material file. DOI: 10.1107/S160053681300946X/hb7066Isup4.mol

Additional supplementary materials:  crystallographic information; 3D view; checkCIF report

## Figures and Tables

**Table 1:** Selected bond lengths (Å)

Ag1—N4^i^	2.354 (3)
Ag1—S2	2.4987 (8)
Ag1—S1	2.5554 (8)
Ag1—S1^ii^	2.6688 (8)

Symmetry codes: (i) 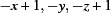; (ii) 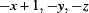.

**Table 2:** Hydrogen-bond geometry (Å, °)

*D*—H⋯*A*	*D*—H	H⋯*A*	*D*⋯*A*	*D*—H⋯*A*
N1—H1⋯N4^i^	0.86 (2)	2.10 (2)	2.954 (4)	171 (3)

Symmetry code: (i) 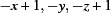.

**Fig. 1.**
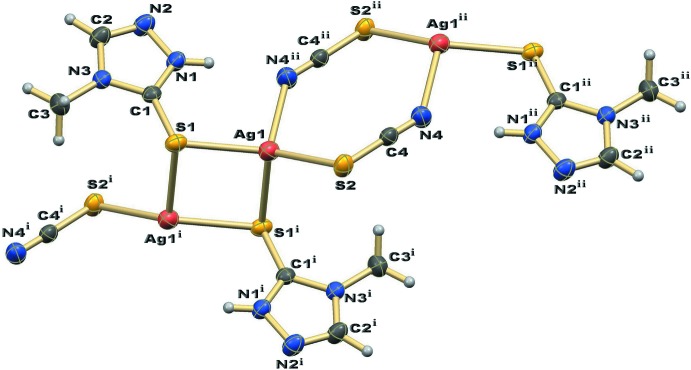
The structure of the title complex with displacement ellipsoids plotted at the 30% probability level.

**Fig. 2.**
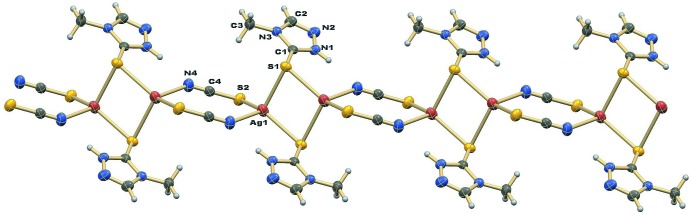
The one-dimensional chain of the title complex.

**Fig. 3.**
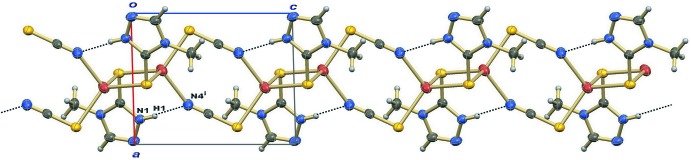
The intra-chain hydrogen-bonding interactions of the title complex.

**Fig. 4.**
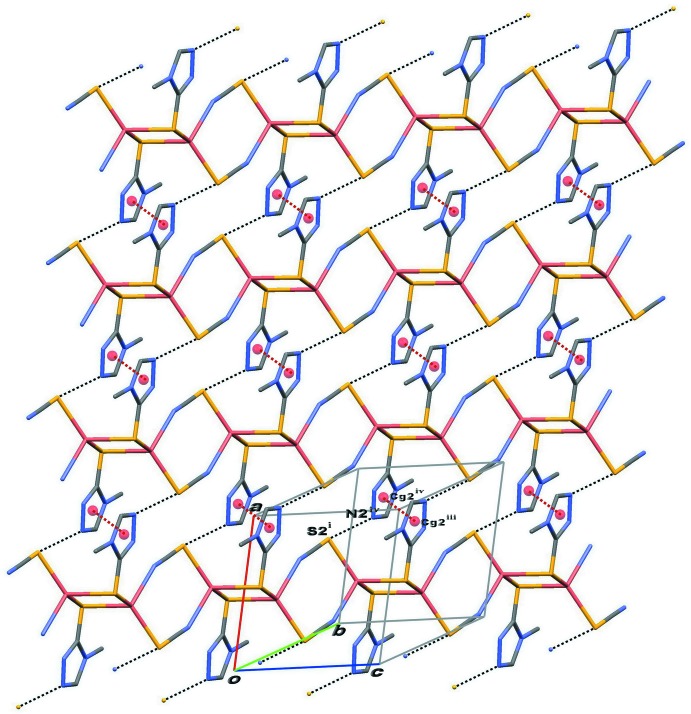
The two-dimensional-layer inter-interactions of the title complex. All H atoms not involving the interactions are omitted.

## supplementary crystallographic information

### Comment

One of the active areas of meterial research is the coordination compounds of the metal ions with the multifunctional ligands leading to the structural diversities and properties (Zhang *et al.*, 2009; Wang *et al.*, 2011). For this work, we report the mixed ligands Ag(I) complex containg multidonor atoms, 4-methyl-1,2,4-triazole-3-thiol (Hmptrz) and thiocyanate ligands. The Hmptrz is one of 1,2,4-triazole derivative ligands - based heterocyclic thioamide containing thiol group which has three potential donor atoms. Both Hmptrz and thiocyanate group are amphidentate ligands, which can bind to the metal center with either the N or S atom or both of them (Zhang *et al.*, 1999; Jiang *et al.*, 2011).

The title complex exhibits a one-dimensional chain polymeric structure and the asymmetric unit consists of one Ag(I) atom, one Hmptrz molecule and one SCN^-^ anion. The chemical structure of this complex is shown in Scheme I and the crystal structure is depicted in Figure 1.

The Ag atom features a distorted tetrahedral environment with the range of angles from 101.00 (2) to 124.52 (3)*^o^*. Each Ag is bonded by two *µ*_2_-S-bridging atoms of two Hmptrz molecules with the distances of 2.5554 (8) and 2.6688 (8) Å. The other two coordination sites are occupied by S and N atoms from the different *µ*_2_-1,3-SCN bridges coordinated as a pair alternating bidentate end to end fashion similar to those complexes of the same thiocyanate bridges (Chen *et al.*, 1999; Diaz *et al.*, 1999; Goher *et al.*, 2000; Song *et al.*, 2000; Cai *et al.*, 2007; Saithong *et al.* 2007). The Cu—S_thiocyanato_ and Cu—N_thiocyanato_ bond distances are 2.4987 (8) and 2.2354 (4) Å, respectively. The SCN bond angle is almost perfectly linear [178.6 (3)°] as compare with the same *µ*_2_-1,3-SCN configuration mode of those complexes (Vicente *et al.*,1997; Song *et al.*, 2000). The S2—C4 and C4—N4 distances [1.646 (3) and 1.150 (4) Å] refer to thiocyanate resonance form which indicate to a π-delocalized system along the metal-thiocyanate chain (Zhang *et al.*, 1999).

An infintite one-dimensional structure of this complex is based on [Ag(*µ*_2-_Hmptrz)(*µ*_2_-1,3-SCN)] double-bridges, in which both Hmptrz and SCN^-^ ligands adopt the *µ*_2_-end-on and *µ*_2_-end-to-end bridging mode, respectively. As illustated in Figure 2, the Hmptrz and thiocyanato ligands interconnect the Ag(I) ions into an infinite chain generated by the unit *c* translation runing parallel to *c* axis, which consist of four-membered ring [—Ag—S—Ag—S—] and eight-membered ring [—Ag—S═C═N—Ag—S═C═N—]. In addition, The Ag···Ag separation with the distances of 3.3241 (5) Å in the four-membered ring is slighty shorter than the sum of the van der Waals radii of Ag atoms (3.44 Å), which indicates that there is the Ag···Ag interaction.

The weak intra-molecular hydrogen bonding interaction [N1—H1···N4^i^, (i) = -*x* + 1, -*y*, -*z* + 1] is found between N(1) of triazole ring and N(4) of thiocyanate bridging ligand at 2.954 (4) Å. The inter-short contact at 3.239 (3) Å arises from S2 donor of triazole ring with N2 acceptor from the thiocyanate bridge of the neighbouring adjacent chain which is smaller than the sum of S and N van der Waals radii (1.80 + 1.55 Å). In addition, the π···π stacking between the triazole rings of the neighbouring chain is observed with the centroid-centriod distance of 4.0762 (18) Å. Both of these interactions generate the supramolecular layer interactions related by *ac-*plane. A view of intra-molecular hydrogen bonding is depected in Figure 3 and The layered network interactions in crystal packing are shown in Figure 4.

### Experimental

A mixture of AgNO_3_ (0.15 g, 0.88 mmol), KSCN (0.09 g, 0.87 mmol) in EtOH 30 ml was heated and stirred to 75 °C for 1 h. After that, the Hmptrz ligand (0.1 g, 0.087 mmol was added to the mixture and further continuous stirring for 12 h. The colorless crystals of the complex were obtained after the colorless filtrate was kept to stand at room temperature for a day. The complex melts at 130–132°C.

### Refinement

All carbon H-atom of the triazole ring and the methyl group were placed in calculated positions (C-*sp*^2^—H = 0.93 and C-*sp*^3^*=* 0.96 Å) and were included in the refinement in the riding-model approximation, with *U*_iso_(H) = 1.2*U*_eq_(C) and *U*_iso_(H) = 1.5*U*_eq_(C), respectively. The H atom of triazole ring N atom is located in a difference map and restrained, N—H = 0.86 Å with *U*_iso_(H) = 1.2*U*_eq_(N).

### Crystal data

**Table:**

[Ag_2_(NCS)_2_(C_3_H_5_N_3_S)_2_]	*Z* = 1
*M**_r_* = 562.22	*F*(000) = 272
Triclinic, *P*1	*D*_x_ = 2.209 Mg m^−^^3^
Hall symbol: -P 1	Mo *K*α radiation, λ = 0.71073 Å
*a* = 7.4842 (6) Å	Cell parameters from 3160 reflections
*b* = 7.5420 (6) Å	θ = 2.5–28.1°
*c* = 8.4262 (7) Å	µ = 2.82 mm^−^^1^
α = 79.985 (2)°	*T* = 293 K
β = 84.329 (2)°	Block, colourless
γ = 64.508 (1)°	0.31 × 0.12 × 0.05 mm
*V* = 422.62 (6) Å^3^	

### Data collection

**Table:**

Bruker APEX CCD diffractometer	2083 independent reflections
Radiation source: fine-focus sealed tube	1904 reflections with *I* > 2σ(*I*)
Graphite monochromator	*R*_int_ = 0.026
Frames, each covering 0.3 ° in ω scans	θ_max_ = 28.3°, θ_min_ = 2.5°
Absorption correction: multi-scan (*SADABS*; Bruker, 2003)	*h* = −9→9
*T*_min_ = 0.682, *T*_max_ = 0.879	*k* = −10→10
5887 measured reflections	*l* = −11→11

### Refinement

**Table:**

Refinement on *F*^2^	Primary atom site location: structure-invariant direct methods
Least-squares matrix: full	Secondary atom site location: difference Fourier map
*R*[*F*^2^ > 2σ(*F*^2^)] = 0.029	Hydrogen site location: inferred from neighbouring sites
*wR*(*F*^2^) = 0.070	H atoms treated by a mixture of independent and constrained refinement
*S* = 1.05	*w* = 1/[σ^2^(*F*_o_^2^) + (0.0262*P*)^2^ + 0.3652*P*] where *P* = (*F*_o_^2^ + 2*F*_c_^2^)/3
2083 reflections	(Δ/σ)_max_ < 0.001
104 parameters	Δρ_max_ = 0.79 e Å^−^^3^
1 restraint	Δρ_min_ = −0.63 e Å^−^^3^

### Special details

**Table:**

Geometry. All e.s.d.'s (except the e.s.d. in the dihedral angle between two l.s. planes) are estimated using the full covariance matrix. The cell e.s.d.'s are taken into account individually in the estimation of e.s.d.'s in distances, angles and torsion angles; correlations between e.s.d.'s in cell parameters are only used when they are defined by crystal symmetry. An approximate (isotropic) treatment of cell e.s.d.'s is used for estimating e.s.d.'s involving l.s. planes.
Refinement. Refinement of *F*^2^ against ALL reflections. The weighted *R*-factor *wR* and goodness of fit *S* are based on *F*^2^, conventional *R*-factors *R* are based on *F*, with *F* set to zero for negative *F*^2^. The threshold expression of *F*^2^ > σ(*F*^2^) is used only for calculating *R*-factors(gt) *etc*. and is not relevant to the choice of reflections for refinement. *R*-factors based on *F*^2^ are statistically about twice as large as those based on *F*, and *R*- factors based on ALL data will be even larger.

### Fractional atomic coordinates and isotropic or equivalent isotropic displacement parameters (Å^2^)

**Table:**

	*x*	*y*	*z*	*U*_iso_*/*U*_eq_	
Ag1	0.43439 (4)	0.06942 (4)	0.18148 (3)	0.06373 (11)	
S1	0.45768 (10)	0.28186 (11)	−0.08435 (10)	0.04902 (17)	
N1	0.8089 (4)	0.2218 (4)	0.0367 (3)	0.0471 (5)	
H1	0.784 (5)	0.168 (5)	0.130 (3)	0.057*	
N2	0.9737 (4)	0.2569 (4)	−0.0040 (3)	0.0580 (7)	
N3	0.7724 (3)	0.3598 (3)	−0.2066 (3)	0.0443 (5)	
C1	0.6839 (4)	0.2840 (4)	−0.0829 (3)	0.0399 (5)	
C2	0.9452 (4)	0.3410 (5)	−0.1505 (4)	0.0548 (7)	
H2	1.0329	0.3844	−0.2120	0.066*	
C3	0.6939 (6)	0.4444 (6)	−0.3672 (4)	0.0614 (8)	
H3A	0.6677	0.3493	−0.4116	0.092*	
H3B	0.7889	0.4779	−0.4352	0.092*	
H3C	0.5733	0.5619	−0.3608	0.092*	
S2	0.13129 (11)	0.14847 (14)	0.36025 (10)	0.0576 (2)	
C4	0.2341 (4)	0.0469 (5)	0.5367 (4)	0.0479 (6)	
N4	0.3024 (4)	−0.0245 (5)	0.6610 (3)	0.0661 (8)	

### Atomic displacement parameters (Å^2^)

**Table:**

	*U*^11^	*U*^22^	*U*^33^	*U*^12^	*U*^13^	*U*^23^
Ag1	0.06481 (18)	0.0881 (2)	0.05807 (16)	−0.04888 (16)	0.01183 (12)	−0.02344 (13)
S1	0.0410 (4)	0.0516 (4)	0.0602 (4)	−0.0237 (3)	−0.0010 (3)	−0.0116 (3)
N1	0.0437 (12)	0.0499 (13)	0.0494 (13)	−0.0240 (11)	−0.0036 (10)	0.0014 (10)
N2	0.0431 (13)	0.0646 (16)	0.0680 (17)	−0.0281 (12)	−0.0086 (12)	0.0049 (13)
N3	0.0451 (12)	0.0457 (12)	0.0454 (12)	−0.0225 (10)	0.0004 (9)	−0.0069 (10)
C1	0.0400 (13)	0.0357 (12)	0.0466 (13)	−0.0172 (10)	0.0015 (10)	−0.0107 (10)
C2	0.0427 (15)	0.0599 (18)	0.0645 (19)	−0.0275 (14)	−0.0007 (13)	−0.0007 (15)
C3	0.073 (2)	0.072 (2)	0.0447 (15)	−0.0374 (18)	−0.0050 (15)	−0.0028 (14)
S2	0.0427 (4)	0.0772 (5)	0.0541 (4)	−0.0308 (4)	−0.0013 (3)	0.0023 (4)
C4	0.0431 (14)	0.0621 (17)	0.0496 (15)	−0.0326 (13)	0.0082 (12)	−0.0135 (13)
N4	0.0619 (17)	0.100 (2)	0.0501 (15)	−0.0486 (17)	−0.0010 (13)	−0.0081 (15)

### Geometric parameters (Å, º)

**Table:**

Ag1—N4^i^	2.354 (3)	N3—C1	1.354 (3)
Ag1—S2	2.4987 (8)	N3—C2	1.363 (4)
Ag1—S1	2.5554 (8)	N3—C3	1.455 (4)
Ag1—S1^ii^	2.6688 (8)	C2—H2	0.9300
Ag1—Ag1^ii^	3.3241 (5)	C3—H3A	0.9600
S1—C1	1.701 (3)	C3—H3B	0.9600
S1—Ag1^ii^	2.6688 (8)	C3—H3C	0.9600
N1—C1	1.325 (3)	S2—C4	1.646 (3)
N1—N2	1.369 (3)	C4—N4	1.150 (4)
N1—H1	0.860 (18)	N4—Ag1^i^	2.354 (3)
N2—C2	1.278 (4)		
			
N4^i^—Ag1—S2	107.73 (7)	C1—N3—C3	125.5 (2)
N4^i^—Ag1—S1	106.91 (7)	C2—N3—C3	127.7 (3)
S2—Ag1—S1	124.52 (3)	N1—C1—N3	104.4 (2)
N4^i^—Ag1—S1^ii^	104.55 (9)	N1—C1—S1	129.2 (2)
S2—Ag1—S1^ii^	110.41 (3)	N3—C1—S1	126.3 (2)
S1—Ag1—S1^ii^	101.00 (2)	N2—C2—N3	112.6 (3)
N4^i^—Ag1—Ag1^ii^	115.17 (8)	N2—C2—H2	123.7
S2—Ag1—Ag1^ii^	135.70 (2)	N3—C2—H2	123.7
S1—Ag1—Ag1^ii^	52.010 (19)	N3—C3—H3A	109.5
S1^ii^—Ag1—Ag1^ii^	48.991 (18)	N3—C3—H3B	109.5
C1—S1—Ag1	104.45 (10)	H3A—C3—H3B	109.5
C1—S1—Ag1^ii^	99.55 (9)	N3—C3—H3C	109.5
Ag1—S1—Ag1^ii^	79.00 (2)	H3A—C3—H3C	109.5
C1—N1—N2	113.0 (2)	H3B—C3—H3C	109.5
C1—N1—H1	122 (2)	C4—S2—Ag1	100.04 (10)
N2—N1—H1	125 (2)	N4—C4—S2	178.6 (3)
C2—N2—N1	103.3 (2)	C4—N4—Ag1^i^	142.0 (2)
C1—N3—C2	106.7 (2)		
			
N4^i^—Ag1—S1—C1	11.91 (13)	C3—N3—C1—S1	−3.5 (4)
S2—Ag1—S1—C1	138.45 (9)	Ag1—S1—C1—N1	−10.7 (3)
S1^ii^—Ag1—S1—C1	−97.15 (9)	Ag1^ii^—S1—C1—N1	−91.7 (3)
Ag1^ii^—Ag1—S1—C1	−97.15 (9)	Ag1—S1—C1—N3	172.3 (2)
N4^i^—Ag1—S1—Ag1^ii^	109.06 (9)	Ag1^ii^—S1—C1—N3	91.3 (2)
S2—Ag1—S1—Ag1^ii^	−124.40 (3)	N1—N2—C2—N3	−0.6 (4)
S1^ii^—Ag1—S1—Ag1^ii^	0.0	C1—N3—C2—N2	1.2 (4)
C1—N1—N2—C2	−0.2 (4)	C3—N3—C2—N2	−178.9 (3)
N2—N1—C1—N3	0.9 (3)	N4^i^—Ag1—S2—C4	−24.14 (14)
N2—N1—C1—S1	−176.7 (2)	S1—Ag1—S2—C4	−150.33 (11)
C2—N3—C1—N1	−1.2 (3)	S1^ii^—Ag1—S2—C4	89.46 (11)
C3—N3—C1—N1	178.9 (3)	Ag1^ii^—Ag1—S2—C4	141.07 (11)
C2—N3—C1—S1	176.4 (2)		

Symmetry codes: (i) −*x*+1, −*y*, −*z*+1; (ii) −*x*+1, −*y*, −*z*.

### Hydrogen-bond geometry (Å, º)

**Table:**

*D*—H···*A*	*D*—H	H···*A*	*D*···*A*	*D*—H···*A*
N1—H1···N4^i^	0.86 (2)	2.10 (2)	2.954 (4)	171 (3)

Symmetry code: (i) −*x*+1, −*y*, −*z*+1.
